# Iron depletion results in Src kinase inhibition with associated cell cycle arrest in neuroblastoma cells

**DOI:** 10.14814/phy2.12341

**Published:** 2015-03-29

**Authors:** Gamini Siriwardana, Paul A Seligman

**Affiliations:** Division of Hematology, University of Colorado School of MedicineAurora, Colorado

**Keywords:** Cell cycle, iron chelation, neuroblastoma, Src kinase

## Abstract

Iron is required for cellular proliferation. Recently, using systematic time studies of neuroblastoma cell growth, we better defined the G1 arrest caused by iron chelation to a point in mid-G1, where cyclin E protein is present, but the cyclin E/CDK2 complex kinase activity is inhibited. In this study, we again used the neuroblastoma SKNSH cells lines to pinpoint the mechanism responsible for this G1 block. Initial studies showed in the presence of DFO, these cells have high levels of p27 and after reversal of iron chelation p27 is degraded allowing for CDK2 kinase activity. The initial activation of CDK2 kinase allows cells to exit G1 and enter S phase. Furthermore, we found that inhibition of p27 degradation by DFO is directly associated with inhibition of Src kinase activity measured by lack of phosphorylation of Src at the 416 residue. Activation of Src kinase occurs very early after reversal from the DFO G1 block and is temporally associated with initiation of cellular proliferation associated with entry into S phase. For the first time therefore we show that iron chelation inhibits Src kinase activity and this activity is a requirement for cellular proliferation.

## Introduction

Iron is required for cellular proliferation, particularly rapidly growing cells including cancer cells (Robbins and Pederson 1970; Rudland et al. 1977; Kontoghiorghes et al. 1986; Le and Richardson 2002). Clinical measurements of iron status in epidemiologic studies have shown a lower incidence of cancer in iron-depleted individuals (Zacharski et al. 2008), better survival in patients whose tumors retain less iron (Pinnix et al. 2010), and a higher incidence in those with or at risk for iron overload (Lenarduzzi et al. 2013; Liu et al. 2013). In the past, it has been suggested that agents that interfere with iron utilization might be used to treat cancer.

Examples of some of these agents used in limited clinical studies of cancer treatment include gallium, a relatively inert metal that inhibits cellular iron uptake (Seligman and Crawford 1991), or the iron chelator deferrioximine (DFO) (Donfrancesco et al. 1992; Helson and Helson 1992; Philip 1992; Brodie et al. 1993) that has also been used for many years as a treatment for iron overload. Besides deferrioxamine, a chelator that has been considered the “gold standard” for treatment of iron overload, new iron chelators are not only more practical (i.e., oral absorption) but have improved iron chelation efficacy, and are lipophilic potentially affecting other biologic processes. Therefore, these agents may well have more potential for cancer treatment including in combination with chemotherapy (Yu et al. 2012; Lui et al. 2013; Potuckova et al. 2014).

As iron is necessary for so many cellular reactions it would not be surprising that it would be required for more than one process necessary for cell division. For example, iron chelation inhibits the iron requiring enzyme ribonucleotide reductase that is necessary for DNA synthesis (Eriksson et al. 1984; Zhang et al. 2011). Besides the early S phase arrest caused by inhibition of ribonucleotide reductase, numerous studies have shown that iron is required for one, or more than one, cellular reaction that allows cells to proceed through G1 phase into G1/S and enter S phase (Brodie et al. 1993; Le and Richardson 2002; Fu and Richardson 2007).

Recently, using systematic time studies of neuroblastoma cell growth *in vitro* with iron chelation treatment, we better defined the G1 arrest to a point in mid-G1, where cyclin E protein is present, but the cyclin E/CDK2 complex activity may be inhibited (Siriwardana and Seligman 2013). In this study, we again used the neuroblastoma SKNSH cell line to better define “upstream” mechanisms responsible for the G1 block. The SKNSH cells are optimal for studies of cell cycle events because the cells are consistently diploid show synchronous G_1_ (or G_0_/G_1_) onset with contact inhibition and uniformly respond to various stimuli including a stimulus for proliferation such as subculture in serum containing media (Siriwardana and Seligman 2013). These conditions allow for minimal changes in the physiology of cellular proliferation or molecular makeup of SKNSH and make the cell line particularly useful for the study of very early cell cycle events that promote cell proliferation.

Using specific antibodies that recognize phosphorylation sites we show that iron is required for CDK2/cyclin E complex activity. Our initial studies showed SKNSH cells in the presence of DFO have high levels of p27 and little or no measureable p21 and p57. In this manuscript we, therefore, show that increased CDK2 kinase activity after reversal of the DFO block is temporally related to downregulation of the Cip/Kip CDK2 inhibitor p27 (Chu et al. 2007). We studied Src kinase as it causes initial phosphorylation and later degradation of p27 and resultant activation of CDK2/E kinase. Src or cellular-Src (cSrc) is a ubiquitous highly conserved protein. Src kinase acts as a fundamental enzyme for cell proliferation, adhesion, migration and tumorigenesis by transmitting extra cellular signals across the cell membrane (Cooper and MacAuley 1988; Harvey et al. 1989; Brown and Cooper 1996; Parsons and Parsons 2004; Cowan-Jacob et al. 2005; Chu et al. 2007; Ingley 2008). For the first time we show that iron chelation specifically inhibits Src kinase activation by inhibiting phosphorylation at the 416 residue (Cooper and MacAuley 1988; Harvey et al. 1989; Cowan-Jacob et al. 2005). We show activation of Src kinase occurs very early after reversal of the DFO G1 block with associated initiation of cellular proliferation measured by cells entry into S phase.

## Materials and Methods

SKNSH (ATCC HTB-II) cells were grown to confluency (contact inhibition) in 10 cm tissue culture dishes in 10 mL RPMI1640 and 10% Fetal Calf Serum (FCS), control median (CM), and subcultured in 3 cm tissue culture plates with 2.5 mL of CM with 100 uM DFO. After incubation for a minimum of 20 h, the cells were released from the DFO block by adding control media, but with heat-inactivated FCS: (reversal media (RM)), and the different experiments described below were conducted.

Changes in cell growth after subculture and under various tissue culture conditions were assesses by cell counts using a cytometer (Brodie et al. 1993). Cell cycle analysis was performed by staining with propidium iodide and analyzed by flow cytometry as previously described (Brodie et al. 1993; Siriwardana and Seligman 2013). For most experiments cell cycle analysis was performed 18–24 h after each timed treatment in order to best synchronize cells at a specific arrest point.

DFO was obtained from CIBA-GEIGY Canada. The DFO stock solution was reconstituted in distilled water to 100 mmol/L DFO and stored at −20°C. Unless otherwise stated, DFO treatments were made at 100 *μ*mol/L dose (Fu and Richardson 2007). Src inhibitor Saracatinib (AZD 0530) and CDK2 inhibitors (BMS265246 and Dinaciclib) were utilized. It is clear from product information that CDK2 inhibitors, inhibit other CDK's to some degree and AZD is not entirely specific just for Src kinase inhibition. Both were obtained from Biotang Inc. (Framingham, Massachusetts) and dissolved into a 10 mmol/L concentration of DMSO. Unless otherwise stated, these treatments were made at 10 *μ*mol/L dose. Antibodies for cyclin A, cyclin E, pP27, p27, and *β*-actin were obtained from Santa Cruz Biotechnology (Santa Cruz, California). Antibodies for Src, pSrc, CDK2, pCDK2 were obtained from Cell Signaling Technology (Menlo Park, California). SKNSH (ATCC HTB-11) human neuroblastoma cell lines were maintained in stock culture in CM.

For immunoblot analysis, cells were grown in 35-mm tissue culture dishes in 2.5 mL of CM. Harvesting of cells after various treatments was carried out by aspirating the growth medium, followed by washing of the cells with cold PBS and adding 200 *μ*L L of SDS sample buffer at 90°C and heating to 80°C for 5 min. Proteins were resolved by subjecting 40 *μ*L L samples to sodiumdodecyl sulfate polyacrylamide gel electrophoresis (SDS-PAGE) (Tyrsted 1982; Siriwardana and Seligman 2013). Proteins were transferred to polyvinylidene difluoride (PVDF) membranes (EMD Millipore, Billerica, MA) in a 192 mmol/L lysine, 25 mmol/L Tris, and 20% methanol for 1.5 h. Filters were blocked by PBS with 0.2% tween 20 and 5% nonfat milk and probed using specific antibodies (Siriwardana and Seligman 2013). The proteins of interest were detected using the enhanced chemiluminescence (ECL) procedure. Uniformity of protein loading was measured as necessary by probing beta-actin protein, after the membrane was stripped (Siriwardana and Seligman 2013). When multiple probes were used on the same membrane, the previous probes were removed by stripping using the stripping buffer (Siriwardana and Seligman 2013). Treatments were made in duplicate during each experiment.

## Results

**Figure 1:**
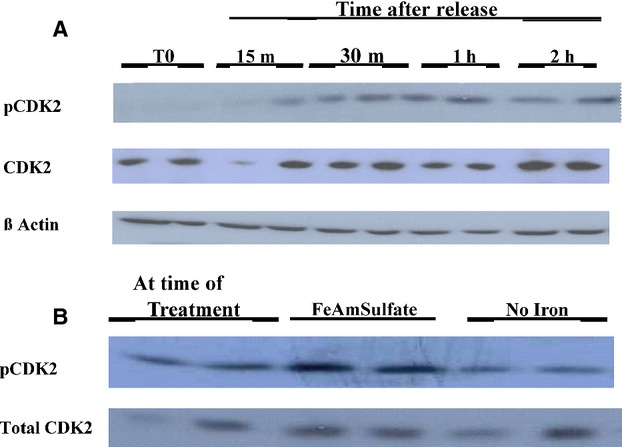
CDK2 is phosphorylated soon after reversal from DFO. (A) Confluent SKNSH cells were subcultured into RPMI/10% FCS (CM) with 100 um DFO and incubated. After 20 h the medium was replaced with new medium containing no DFO and 10% heat deactivated FCS (RM). The cells were harvested at regular intervals beginning at 15 min after RM added, the medium was aspirated and hot SDS loading buffer was added. Westerns were performed as described and pCDK2 levels were determined. Thereafter, the blot was stripped and probed for total CDK2 and then B Actin. Each treatment was conducted in duplicate. (B) Confluent SKNSH cells were subcultured in CM with 100 *μ*m DFO and incubated. After 20 h two plates were treated with Iron (II) Ammonium sulfate hexahydrate to 300 *μ*mol/L and two plates continued in CM with DFO (without added iron). One hour later cells were harvested by aspirating the medium and treated with hot SDS loading buffer. Westerns were performed as described and pCDK2 levels were determined. Thereafter, the blot was stripped and reprobed for total CDK2.

Because activation of CDK2 on the CDK2/cyclin E complex is necessary for transition from G1 phase into G1/S, (Ganoth et al. 2001) we measured phosphorylation of CDK2 in SKNSH neuroblastoma cells. After SKNSH cells are released from the G1 block caused by iron chelation (DFO treatment), there is significantly increased phosphorylation of CDK2 starting at about 15 min after reversal, this increase continues for about 1–2 h and levels off or decreases at 2–4 h (Fig.1A and data not shown). The activation (phosphorylation) of CDK2 allows for synthesis of specific proteins for cells to proceed through G1/S phase and enter S phase. In other experiments some cells subcultured in DFO with added excess Fe as ferrous ammonium sulfate for 1 h showed a marked increase in pCDK2 compared to cells with no added iron (Fig.1B).

### Iron chelation inhibits p27 degradation

**Figure 2:**
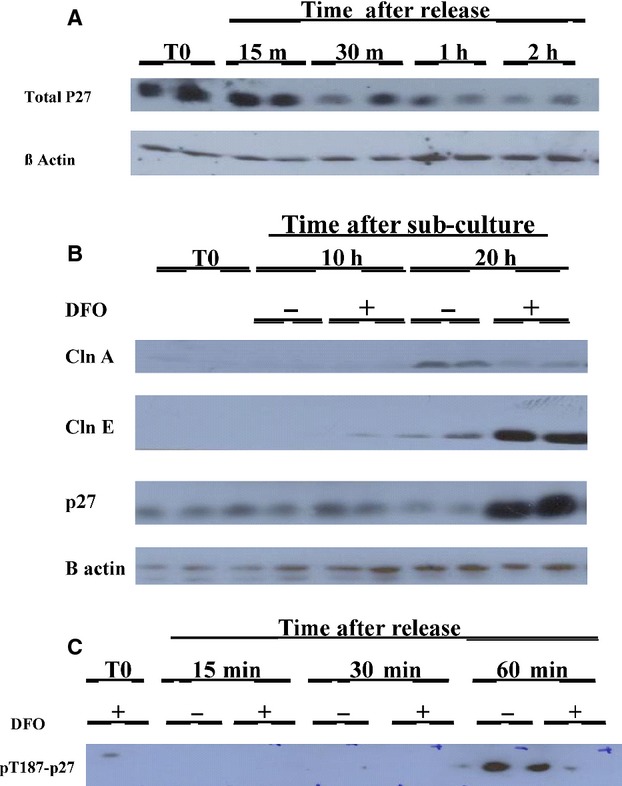
(A) Total p27 levels decrease rapidly after release from the DFO block. Confluent SKNSH cells were subcultured into RPMI/10% FCS with 100 *μ*m DFO and incubated. After 20 h the medium was replaced with new medium as RM. The cells were harvested at regular intervals beginning 15 min after aspirating the medium and adding hot SDS loading buffer. Westerns were performed as described and (total) p27 levels were determined. Thereafter, the blot was stripped and probed for B Actin. Each treatment was conducted in duplicate. (B) p27 accumulation occurs in cells subcultured in DFO at the same time as cyclin E accumulation. Confluent untreated SKNSH cells were subcultured into RPMI/10% FCS with or without 100 *μ*m DFO. The cells were harvested at 10 h and 20 h by aspirating the medium and treating with hot SDS loading buffer. Westerns were performed as described. First the levels of cyclin A were determined. Following this the blot was probed for cyclin E, p27, and *β*-actin with the blot stripped after each probe. (C) Although it was unstable, some experiments showed that p27 kip is phosphorylated at thr187 following reversal from DFO. Confluent SKNSH cells were subcultured into RPMI/10% FCS with 100 *μ*m DFO and incubated. The medium was replaced with RM and with or without DFO. The medium was aspirated at the times indicated in the figures and westerns were performed, for py187p27 levels.

Inhibitors that bind to the CDK2 complex include p27 (Hengst and Reed 1996; Bloom and Pagano 2003). As the above studies indicated that inhibition of the cyclin E/CDK2 complex occurred with iron chelation we measured total p27 protein levels and p27 phosThr187 (Fu and Richardson 2007). Progressive phosphorylation of p27 leads to its degradation and facilitates the activation of CDK2 (Hengst and Reed 1996; Bloom and Pagano 2003) and cells then proceed through G1/S. As shown in Figure2, measurements of total p27 were relatively high in DFO-treated confluent cells but within 30 min after subculture in RM, p27 decreased (Fig.2A). Untreated cells subcultured in DFO show accumulation of total p27 as compared to cells subcultured in CM at 20 h. This change with DFO treatment occurs at about the same time as cyclin E accumulation as compared to cyclin A expression seen in untreated cells (2B). Since it was difficult to measure because it was unstable, phosphorylated p27 at Thr 181 was measured in only a few experiments in cells subcultured to RM (Fig.2C).

### An early event inhibited by iron chelation is activation of Src by phospholation at tyrosine 416

**Figure 3:**
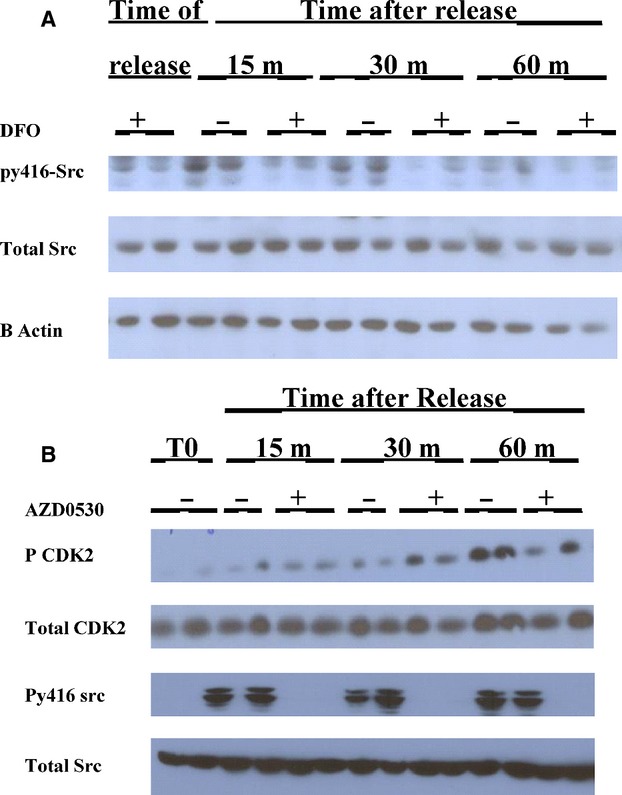
Src 414 phosphorylation is inhibited in the presence of DFO (A) Confluent SKNSH cells were subcultured into RPMI/10% heat-inactivated FCS with 100 *μ*m DFO and incubated. After 20 h the medium was replaced with new medium containing no DFO and 10% heat deactivated FCS. The cells were harvested at regular intervals beginning 15 min after aspirating the medium and adding hot SDS loading buffer. Westerns were performed as described and Src p416 levels were determined. Thereafter, the blot was stripped and probed for B Actin. Each treatment was conducted in duplicate. Based on densiomitry, oSrc/B-Actin (average of duplicates) for RM versus RM with DFO, respectively. 0 time 0.35 versus 0.29; 15 min 0.78 versus 0.28; 30 min 0.55 versus 0.21; 60 min 0.31 versus 0.26. (B) Src kinase inhibitor AZD 0503 inhibits CDK2 phosphorylation in SKNSH cells. SKNSH cells were subcultured in CM, 20 h later half of the plates received added AZD0503 to a 10 *μ*mol/L concentration and were left for 30 min. Thereafter, the medium in all plates was replaced with new RM. The plates that were preincubated with the drug received RM with AZD0503, whereas the other half received no treatment. The cells were harvested using hot SDS buffer at the times given in the Figure. Westerns were probed first for py416Src, then total Src, and lastly B-Actin.

Activated Src has been shown to initially phosphorylate p27 (Chu et al. 2007) enabling the activation of the CDK2 complex. Activated Src is an early event after a stimulus for proliferation. We, therefore, measured Src activation using specific antibodies to detect phosphorylation Src at tyrosine 416. Figure3 shows the detection of phosphorylated Src after cells are preincubated with DFO and replaced with either RM or RM containing DFO (3A). When 100 *μ*m iron (II) Ferrous ammonium sulfate was added to the RM there was an almost identical increase in Src p416 at 15 min, (data not shown) indicating the iron present in the RM, without added iron, was sufficient to neutralize the trace DFO left in the washed cells, and the results were not skewed by a marked excess of iron (see Discussion). Activated Src occurs very early in RM-treated cells without DFO (at 15 min) but does not change with cells continuing in DFO. Total Src does not change in either treated or untreated cells, indicating that iron chelation has inhibited phosphorylation of Src at Tyrosine 416. Although phosphorylation of Src at 416 was seen in cells incubated with RM in multiple experiments at 15 min, decreases in Src p416 to minimal levels varied from 30 min to 2 h.

As noted above Cyclin D and p21 levels were not affected by DFO treatment, and in other experiments, the Akt/p13 pathway as well as HIF1 levels were also not affected at these early time points (data not shown).

Adding the Src inhibitor, ADZ0530, into RM after 30 min preincubation in the CM was associated with inhibition of phosphylation of CDK2 after reversal from the DFO arrest (3B). As expected this event is later than inhibition of Src p416 by the added ADZ (3B).

### Cell cycle results with CDK2 inhibitors

**Figure 4:**
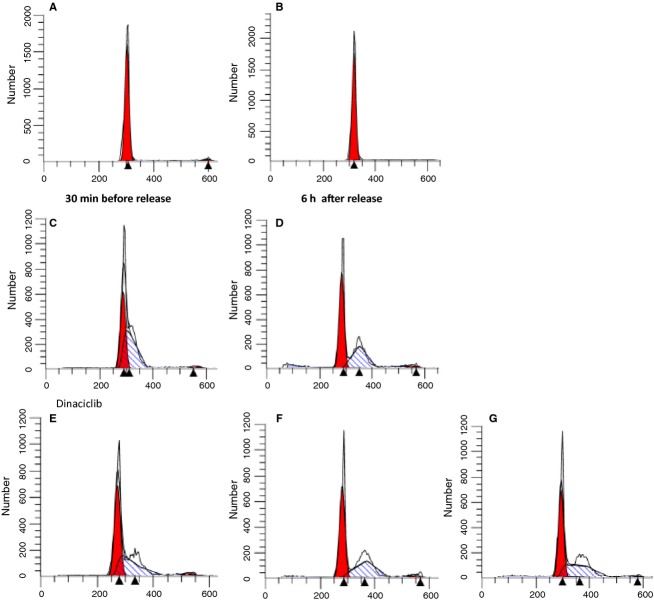
Inhibition of CDK2 activity following reversal from the DFO G1 block prevents cells from proceeding to mitosis. SKNSH cells were incubated overnight with 100 *μ*mol/L DFO in CM. Twenty hours later the cells were released from the DFO block by adding 1 mL of RM. Pretreatments with CDK2 inhibitors were conducted by adding the inhibitors 30 min prior to and along with the RM. Post CDK2 block was carried out by adding the CDK2 inhibitors 6 h after reversal from the DFO block. The cells were further incubated for 18–24 h in the RM, with or without additions, to synchronize cells at a specific block. At that time cells were harvested and FACS analyses were conducted. (A) Time of subculture (B) Time of reversal from DFO (C) BMS added 30 min before reversal (D) BMS added 6 h after reversal (E) Dinaciclib added 30 min before reversal (F) Dinaciclib added 6 h after reversal (G) DFO readded 6 h after reversal (in absence of the CDK2 inhibitor).

Cells subcultured from confluence, in DFO were placed in RM. CDK inhibitors that mainly affect CDK2 as well as other CDK's were added before reversal and 6 h after reversal from the DFO block; 24 h later the cells were assessed for cell cycle using DNA staining with propidium iodide (Fig.4). Figure4 shows that cells continued in DFO for 24 h after subculture show 85% of cells in G1 (4A and 4B). The CDK2 inhibitor BMS (265246) added just before reversal from the DFO G1 block showed accumulation of cells in G1/S and early S phase. If the inhibitor was added after reversal, cells were mainly arrested in S phase but with a shift to mid to late S phase (Fig.4C and D). Cells that were pretreated with dinaciclib, another CDK2 inhibitor before reversal showed 52% of cells in G1 but 44% in early S phase (4F). However, if the CDK2 inhibitor was added 6 h after reversal, as with BMS, there was an obvious shift of cells from early S phase to mid-S phase. An almost identical profile was seen when cells had DFO added 6 h after initial reversal (4G).

### Cell cycle results with Src inhibition

**Figure 5:**
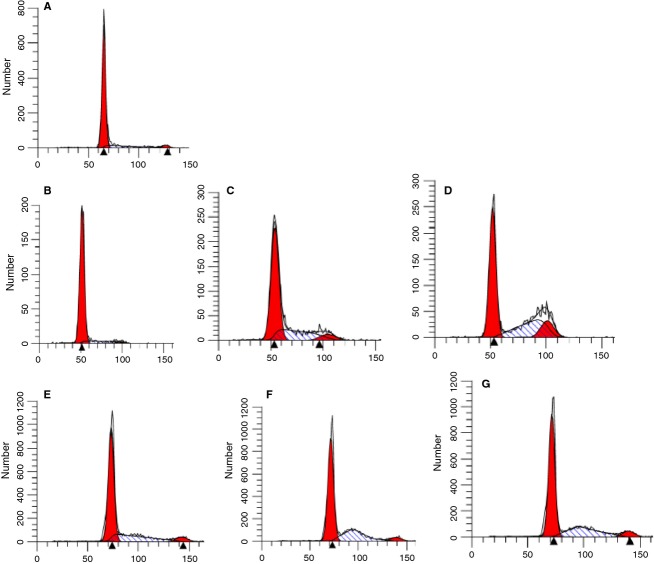
Src is involved in the progression of cells toward mitosis following reversal from the early block by DFO. SKNSH cells were plated in 24-well plates with CM with DFO. Twenty hours later the cells were released from the DFO block by adding 1 mL of RM and FACS was conducted at 18 h after reversal detailed below. (A) AZD 0530 was added 30 min prior to reversal (B) AZD was added 15 min after reversal (C) AZD added 30 min after reversal (D) AZD added 60 min after reversal (E) DFO was readded 15 min after reversal (with no added AZD) (F) DFO was readded 2 h after reversal (G) DFO was readded 4 h after reversal.

Figure5A shows the results using AZD0530, a potent Src inhibitor used clinically in cancer treatment (Navarra et al. 2010). Cells pretreated with AZD before reversal (5A) showed a profile almost identical to continued treatment with DFO 24 h later, and agree with the findings of Src phosphorylation shown in Figure3A. However, if the AZD was added as soon as 15 min after reversal cells were able to enter S phase (5B) and then entered G2/M by 1 h (5C). These results are similar to the profile seen when DFO is added 15 min after reversal (5D) but because of the S phase arrest point caused by DFO cells remain arrested in S phase at 2 h and 4 h after the addition of DFO after reversal (5E, 5F).

## Discussion

Iron utilization to support cellular proliferation has been well studied (Robbins and Pederson 1970; Rudland et al. 1977; Kontoghiorghes et al. 1986; Blatt et al. 1988; Hoyes et al. 1992; Le and Richardson 2002; Chaston et al. 2003; Nurtjahja-Tjendraputra et al. 2007). As might be expected most studies of the mechanism by which iron is required for cellular proliferation have focused on cancer cell lines (Blatt et al. 1988; Hoyes et al. 1992; Chaston et al. 2003; Nurtjahja-Tjendraputra et al. 2007). However, normal cells that proliferate also require iron; for example, besides the obvious need for iron necessary for hemoglobin production, iron is required for erythroid proliferation and is an essential requirement for erythropoietin to promote erythropoiesis (Bullock et al. 2010; Kanbay et al. 2010).

Prior studies have focused on the two main cell cycle events that require iron (Eriksson et al. 1984; Zhang et al. 2011): (1) DNA synthesis that must be maintained by the iron requiring enzyme ribonucleotide reductase; (2) an iron requirement necessary for cells to proceed through G1 phase and enter S phase, therefore causing cells to arrest in G1 (Brodie et al. 1993; Le and Richardson 2002; Wang et al. 2004; Fu and Richardson 2007; Seguin et al. 2011).

In our recently published study, we have provided further experimental evidence that allow us to separate the DNA synthetic block from the G1 block caused by iron depletion (Siriwardana and Seligman 2013). The G1 arrest point is associated with accumulation of cyclin E protein indicating that the block is after “start” but before the accumulation of cyclin A protein that occurs in late G1 (or G1/S) phase (Siriwardana and Seligman 2013). In this manuscript, we confirmed that the G1 block is associated with inhibition of CDK2 activity (phosphorylation). This effect is correlated with lack of p27 phosphorylation so that it remains bound to CDK2 maintaining CDK2 inactivation. p27 accumulation and inhibition of cell entrance into S phase has been described previously as a consequence of DFO treatment of fibroblasts (Wang et al. 2004).

In our investigations of earlier events associated with a stimulus for cellular proliferation we studied Src phosphorylation. We found that under our experimental conditions that a marked increase in Src phosphorylation at the 416 residue occurs rapidly (within 15 min) after cells are subcultured at lower density and exposed to growth medium. Iron chelation by DFO that acts to deplete intacellular iron, inhibits Src phosphorylation at the 416 residue, and inhibits kinase activity as measured by inhibition of cell cycle changes.

Src protein will associate with the cytoplasmic part of the cell membrane (Brown and Cooper 1996; Parsons and Parsons 2004; Chu et al. 2007; Ingley 2008). After receiving a multitude of transmitted extracellular signals activated cSrc kinase acts as an initiator for numerous cascades responsible for cell proliferation, adhesion, migration and/or tumorigenesis (Brown and Cooper 1996; Parsons and Parsons 2004; Chu et al. 2007; Ingley 2008; Gottlieb-Abraham et al. 2013). Under the experimental conditions described above, we are able to study SKNSH cell proliferation as a functional process directly associated with Src kinase activation.

cSrc has a SH_2_ and a SH_3_ (Src homology 2 and homology 3, respectively) closest to the cytoplasmic part of the cell membrane that regulate the kinase activity associated with the N terminus. Phosphorylation of the tyrosine 508 controlled at least in part by Csk results in folding of the SH_2_ domain to the kinase domain inhibiting kinase activity (inactive state) (Cooper and MacAuley 1988; Harvey et al. 1989; Brown and Cooper 1996; Ganoth et al. 2001; Parsons and Parsons 2004; Cowan-Jacob et al. 2005; Chu et al. 2007; Ingley 2008; Okada 2012). The inactive state is dominant for most cells, however, a stimulus for a multitude of cellular processes, particularly in cancer cells (Sen and Johnson 2011), results in kinase activation.

Activation of cSrc kinase is accomplished by several factors and events, including cell surface stimuli discussed above. This activity may be also reflected in other kinases with homology to the cSrc protein called SFK (Src family kinases), SFK are distributed unequally based on tissue type (Parsons and Parsons 2004; Ingley 2008).

There have been some suggestions in the recent literature that iron may be important for Src function such as Src homologues that transport iron in microorganisms (Hsueh et al. 2013; Lau et al. 2013). In eukaryotic cells, Src phosohorylation of transferrin receptor increases breast cancer cell survival apparently unrelated to the receptor iron transport function (Jian et al. 2011). Also, some examples of tyrosine modification enhances binding of metal irons (Baldwin et al. 2008). A more recent study using isolated human proteins and polypeptides presents evidence that specific iron binding to the cSrc COOH domain, if in the inactive state, facilitates activation of kinase activity (Baldwin et al. 2014). This iron requirement for kinase activity when Src is in the inactive (phosphorylated COOH domain) was more directly measured in Lyn, a SFK mostly found in hematopoietic cells (Ingley et al. 2006; Baldwin et al. 2014).

Activation of kinase activity after conformational changes often due to transmembrane transmission can be accomplished by numerous reactions that phosphorylate the active site by “auto phosphorylation” (Cooper and MacAuley 1988; Frame 2002; Cowan-Jacob et al. 2005) as the process of donation of phosphate by various molecules including constitutive phosphates (ATP/ADP) (Weigand et al. 2012) or growth factor receptor kinases and/or other membrane associated proteins that act as receptor tyrosine kinases (Parsons and Parsons 2004). The most specific Src kinase inhibitors mainly function as ATP-binding inhibitors (Navarra et al. 2010; Sen and Johnson 2011). We assessed kinase activation associated with rapid reversal of the iron chelation effects of DFO. Under these experimental conditions SKNSH cells would be stimulated to proliferate, starting at the DFO G_1_ arrest point, rapidly followed by events associated with reversal of this block caused by iron chelation.

Cell cycle experiments using specific inhibitors of cSrc kinase and CDK2 complex kinase activity were compared to the iron chelation (DFO) effects. We were able to demonstrate the following (1) Even though the exact mechanism is different, Src kinase inhibition when added just before reversal of iron chelation showed a similar G1 arrest in cells maintained in DFO without added iron; (2) Reversal of iron chelation results in rapid phosphorylation of cSrc within 15 min (Fig.3). If the cSrc kinase inhibitor is added as early as 15 min after reversal rather than before reversal, cells are able to enter S phase. This effect is seen in cells treated with DFO 15 min after reversal (in effect reversing the reversal). If the cSrc inhibitor is added 1 h after reversal the cells can enter late S and early G2/M phase. However, cells treated with readded DFO 15 min to 6 h after reversal do enter S phase but do not progress through and enter G2/M, indicating that cells are exhibiting the S phase DFO block; (3) cells treated with a CDK2 inhibitor before reversal show what appears to be a block in G1, G1/S and very early S phase. When the CDK2 inhibitor is added 6 h after reversal cells have progressed into late S phase, exhibiting a similar profile to cells with added DFO 6 h after reversal. These data confirm that CDK2/cyclin E or more importantly CDK2/cyclin A kinase activity, as well as iron are necessary for progression through S phase into G2/M, even though the mechanisms for this inhibition are different.

The DFO inhibition of cSrc kinase activity necessary for cellular proliferation is supported by the gel and cell cycle studies presented above. When this effect is reversed cells proceed through G1/S and enter S phase indicating that iron is necessary for Src kinase activity as an initial event for cell proliferation. Src kinase activity necessary for initiation of proliferation then results in downstream events: (1) rapid phosphorylation, and degradation of p27; (2) resultant CDK2 kinase activity; (3) entry into S phase. The activation of Src kinase and CDK2 kinase supported by western blot are temporally supported by functional evidence for cell cycle progression using DNA cell cycle measurements.

Based on our prior studies SKNSH neuroblastoma line appears to be relatively resistant to DFO effects on inhibition of cyclin D synthesis (Rader et al. 2013; Siriwardana and Seligman 2013) shown in studies using other cell lines (Nurtjahja-Tjendraputra et al. 2007). The later block described in SKNSH, that is, inhibition of cyclin E/CDK2 complex activity, would still be a result of inhibition Src kinase activity as Src kinase is responsible for initial phosphorylation allowing for resultant degradation of p27. All the differences noted above with changes in cell cycle protein caused by iron chelation including changes in p27 compared to p21, (Fu and Richardson 2007; Gottlieb-Abraham et al. 2013) whether based on cell type or, less likely experimental design, would still occur with inhibition of Src kinase activity. This inhibition can lead to a common hypothesis as to how divergent findings on “downstream” events caused by iron chelation can be reconciled.

Besides cellular proliferation, the multitude of other events facilitated by Src kinase activation such as cell migration and adhesion may be affected under these experimental conditions. However, these latter events may be measured at a molecular level but from a functional standpoint cell migration and adhesion are difficult to measure at these early time points. Also Src activation measured only by phosphorylation of the 416 residue may be inaccurate particularly when phosphorylation of this site may occur in the closed or inactive position (Irtegun et al. 2013) or other cell membrane events similar to Oubain exposure and the uncertain effect these changes would have on Na/k ATPase effects on Src autophosphorylation that results in kinase activity (Gable et al. 2014). Some of these events may be responsible for the background level of p416Src sometimes seen in cells treated with DFO, but would not explain the rapid change in p416 Src measurements after reversal of the DFO block, particularly when temporally associated with entry into S phase.
